# Effects of Dietary Carbohydrases on Fecal Microbiome Composition of Lactating Sows and Their Piglets

**DOI:** 10.4014/jmb.2203.03026

**Published:** 2022-05-04

**Authors:** Jeong Jae Lee, Minho Song, Hyunjin Kyoung, Kyeong Il Park, Sangdon Ryu, Younghoon Kim, Minhye Shin

**Affiliations:** 1 Institute of Agricultural Science and Technology, Kyungpook National University, Daegu 41566, Republic of Korea; 2 Division of Animal and Dairy Science, Chungnam National University, Daejeon 34134, Republic of Korea; 3 Department of Agricultural Biotechnology and Research Institute of Agriculture and Life Sciences, Seoul National University, Seoul 08826, Republic of Korea; 4 Department of Microbiology, College of Medicine, Inha University, Incheon 22212, Republic of Korea

**Keywords:** Multigrain carbohydrases, microbiome, 16S rRNA gene sequencing

## Abstract

Corn-soybean meal diets are commonly used in the pork industry as a primary source of energy and protein. However, such a diet generally contains non-starch polysaccharides (NSPs) which present a challenge in finding ways to improve their availability and digestibility. Dietary multi-carbohydrases (MCs) have been proposed as an efficient approach to utilize NSPs, and can result in improved growth performance and host intestinal fitness. In this study, we evaluated the effects of MC in lactation diets on gut microbiota composition of lactating sows and their litters. The experimental design contained two dietary treatments, a diet based on corn-soybean meal (CON), and CON supplemented with 0.01% multigrain carbohydrases (MCs). Sow and piglet fecal samples were collected on days 7 and 28 after farrowing. Based on the results from 16S rRNA gene amplicon sequencing, MC led to changes in species diversity and altered the microbial compositions in lactating sows and their piglets. Specifically, the MC treatment induced an increase in the proportions of *Lactobacillus* in piglets. *Clostridium* and *Spirochaetaceae* showed a significantly reduced proportion in MC-treated sows at day 28. Our results support the beneficial effects of dietary carbohydrases and their link with improved production due to better host fitness outcomes and gut microbiota composition.

## Introduction

Corn-soybean meal is the primary source of energy and protein in swine diets. A diet of corn-soybean generally contains non-starch polysaccharides (NSPs), including arabinoxylans, xylans, phytate, glucans and cellulose, which cannot be digested in the small intestine of pigs due to a lack of the digestive enzymes needed to degrade the nutrients [1]. Accordingly, these NSPs negatively affect energy and protein digestibility resulting in decreased growth performance [2, 3].

Researchers in the swine industry have developed various approaches for improving and optimizing the availability and digestibility of NSPs. The addition of multi-carbohydrase (MC), an enzyme mixture consisting of xylanase, β-glucanase, and cellulose, has been suggested as a potentially efficient approach to improve NSP availability [4]. MC breaks down carbohydrates into digestible sugar forms and provides a way for pigs to improve their digestion and absorption of nutrients by reducing the anti-nutritional effects related to NSPs in swine diets [5].

The digested carbohydrates can also be good sources of fermentable nutrients for gut microflora in pigs. The gastrointestinal tract of pigs is colonized with a highly diverse microbiota having a considerable impact on an animal’s immune response, gut physiology, and development [6-8]. The most abundant bacterial genera among swine gut microbiota are gram-positive, strict anaerobes belonging predominantly to *Streptococcus*, *Lactobacillus*, *Eubacterium*, *Clostridium*, and *Peptostreptococcus*. Gram-negative bacteria account for a relatively small portion of the total flora, including *Bacteroides* and *Prevotella* [9]. This microbiota composition can be dynamically changed depending on the available nutrients, especially based on the type and amount of dietary fiber in the colonic digesta [10, 11]. Bedford and Apajalahti proposed that addition of exogenous enzymes such as MCs would improve animal performance not only by interacting with the intestinal microflora but also by providing substrates for microbial fermentation [12].

In a previous study, we showed that addition of dietary MC in the diet of lactating sows improved the productive performance of sows and their litters and modulated their immune response [4]. Considering the importance of intestinal microbiota with respect to the availability of dietary fiber, we hypothesized that the performance improvement may be highly associated with modification of the intestinal microbiota composition. Therefore, in the current study, we evaluated the effects of exogenous carbohydrases in the lactating sow diet on the changes in intestinal microbiota and individual strain composition.

## Materials and Methods

### Experimental Design, Animals, and Diets

A total of 12 lactating sows (Landrace x Yorkshire x Duroc; 218.37 ± 5.5 kg of average BW; 2.0 of average parity) were used in this experiment. Sows were randomly allotted to 12 pens of farrowing crates equipped with an individual feeder and drinker in an environmentally controlled farrowing room. On day 114 of gestation, sows were randomly assigned to 2 dietary treatments. Dietary treatments were a typical lactation diet based on corn and soybean meal (CON) and CON added with 0.01% of dietary multigrain carbohydrase (MC). The MC (DSM Nutrition Korea Ltd., Korea) contained xylanase (2,700 units/g), glucanase (700 units/g), and cellulase (800 units/g). The dietary treatments were formulated to meet or exceed the nutrient requirement estimates of lactating sows [4]. Sows were fed 3.0 kg of the dietary treatments from farrowing until weaning. Sows were given *ad libitum* access to diets and water.

### Sample Collection

Fecal samples of sows were collected on day 7 and 28 of lactation, and fecal samples of their litters were also collected on the same days. The samples of feces were collected from three randomly selected sows in each group and three of their randomly selected piglets by rectal palpation.

### DNA Extraction

After collection, the fecal samples were stored at –20°C until analysis, at which time fecal samples (5.0 g) were bathed in Ringer’s solution (Oxoid, UK) and homogenized in a stomacher for 2 min. Also, 1.0 ml subsample solutions were centrifuged at 15,000 g, at 4°C. The genomic DNA was extracted from aliquots using a Fast DNA Spin Kit (MP Bio 1052 Laboratories, USA).

### PCR Amplification and Sequencing

Each DNA sample was adjusted to a concentration of 1 ng/μl and subjected to PCR according to the 16S Metagenomic Sequencing Library protocols (Illumina, USA). The V4 region of the 16S rRNA genes (primer set: forward, 5′-CCT ACG GGN GGC WGC AG-3′; reverse, 5′-GAC TAC HVG GGT ATC TAA TCC-3′) were analyzed using the Illumina MiSeq platform (Illumina). After measuring the concentration of the index PCR products using PicoGreen (Invitrogen, USA), equimolar PCR amplicons were pooled and sequenced using the MiSeq Reagent Kit v3 (600 cycles) for 301 paired-end bases, following the manufacturer’s protocol based on the MiSeq system platform (Macrogen, Korea). The sequencing results were received in fastq file format.

### Metagenomic Analysis

Fastq files obtained from MiSeq paired-end sequencing data were analyzed using the Mothur (v. 1.41) [13]. In Mothur, reads were merged using the make.contig command, and quality-filtered by the screen.seqs command. We aligned the sequences to the SILVA database v. 138, and the chimeric sequences were removed using the VSEARCH program v2.11.1 [14, 15]. Taxonomic classification was analyzed using the Greengenes-formatted database 14 released in 2013. Moreover, Chloroplast, Archaea, Mitochondria, and Eukaryota sequences were removed from the dataset. Low-abundance operational taxonomic units (OTUs) and singletons were removed using the Mothur subroutine “split.abund” 15, and the OTUs were classified using the distance 0.03 calculation (97% sequence similarity). OTUs and the taxonomy table from Mothur were further analyzed on the R platform v. 3.6.2 using the Phyloseq and Vegan packages (https://github.com/vegandevs/vegan). Analysis of similarities (ANOSIM) was conducted using the vegan package.

### Statistical Analysis

Results were expressed as mean ± SD. The data were analyzed by Student’s *t*-test using GraphPad Prism 9 (USA). Statistical significance was considered at *p*-value < 0.05.

## Results and Discussion

Exogenous enzymes in swine diets improve the performance of growing pigs, especially in relation to factors such as NSP degradation, nutrient transit time, and subsequent changes to the gut microbial population [16]. The gastrointestinal tract of lactating piglets is particularly prone to being greatly affected by maternal nutritional status [17]. Previously, we showed that sows fed MC had less body weight loss, fewer white blood cells (WBC), and lower TGF-β1 expression during lactation than those fed CON [4]. In addition, piglets from sows fed MC had higher average weight gain, less diarrhea, fewer WBC and TGF-β1 expression, and higher IgG and IgM concentration than those from sows fed CON, indicating the beneficial effects of MCS on the productive performance and immune response of lactating sows and their piglets.

### Dietary Carbohydrases Lead to Changes in Species Diversity in Lactating Sows and Piglets

To test our hypothesis that addition of dietary MC in corn-soybean meal diets would modify the fecal bacterial communities in lactating sows and their piglets, we investigated the fecal microbiome compositions of lactating sows assigned to two dietary treatments (CON+MC) and their piglets at days 7 and 28 during lactation. The effects of MC on the fecal microbiota of sows and piglets were evaluated with 16S rRNA gene amplicon sequencing (Table 1). A total of 165,989 bacterial sequencing reads and an average of 27,665 sequencing reads per sample were obtained from all fecal samples. Fecal samples’ coverage in samples was 99-100%, indicating that the majority of microbial phylotypes were detected.

Overall, while the total number of OTUs was slightly lower in lactating sows fed MC compared with the CON group, the value was relatively higher in piglets with MC treatment than the control. Bacterial diversities and richness estimated by Chao1, Shannon, and Simpson diversity indices indicated a similar level of alpha diversity metrics between the MC and CON groups, but the values were lower in the sows fed MC and higher in their piglets compared with their littermates fed CON, respectively.

**Fig. 1:**
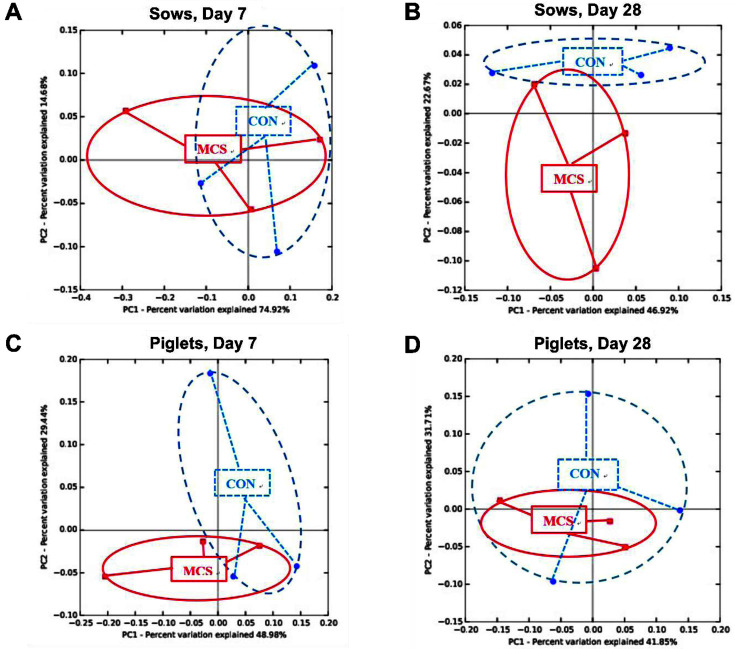
Discriminant analysis of principal components (DAPC) among fecal samples of sows (A and B) and piglets (C and D) at day 7 (A and C) and day 28 (B and D). The 6 differentially abundant bacterial genera represent the number of variables in the model. Individual pig samples for treatments are designated with the following symbols: CON (blue) and MC (red).

We further conducted the discriminant analysis of principal components (DAPC) to identify and describe clusters of microbially associated individuals (Fig. 1). Despite a widespread clustering within the samples, the fecal microbiome profiles of lactating sows and their piglets were distinct between the MC and CON groups, especially in the groups of sows at day 28 during lactation (R = 0.2889 and significance < 0.01 by ANOSIM).

The potential effects of dietary carbohydrases have been pointed out by a number of reports, in which it was indicated that feed enzymes might influence the intestinal microbiota by reducing the amount of undigested substrates or by creating short-chain oligosaccharides as prebiotics from the NSP content in the cell wall [18]. Hübener *et al*. reported that supplemental NSP-degrading carbohydrases such as xylanase and β-glucanase changed the composition and metabolic potential of bacterial populations in the intestine [19]. The microbial diversity in piglets was altered differently compared to the sows, which could be explained by the fact that the gastrointestinal tract of a neonatal piglet is maternally independent and affected by early-life environment factors [20]. These results suggest that MC treatment in the feed led to bacterial species diversity, while the sows and their piglets were influenced differentially during the process.

### Dietary Carbohydrases Alter Microbial Compositions at the Phylum Level in Lactating Sows and Piglets

According to the OTU assignment, an average of 86.5% of the OTUs was assigned to 126 genera (Fig. S1). Thirty-eight genera including *Akkermansia*, *Bacteroides*, *Bifidobacterium*, *Campylobacter*, *Clostridium*, *Desulfovibrio*, *Escherichia*, *Lactobacillus*, *Prevotella*, and *Ruminococcus* were commonly present in sows and piglets. Several genera such as *Gordonibacter* and Mycoplasma were only found in sows, while some bacteria including *Eggerthella*, *Howadella*, *Sutterella*, *Anaerovibrio*, and *Enterorhabdus* were only detected in piglets.

**Fig. 2:**
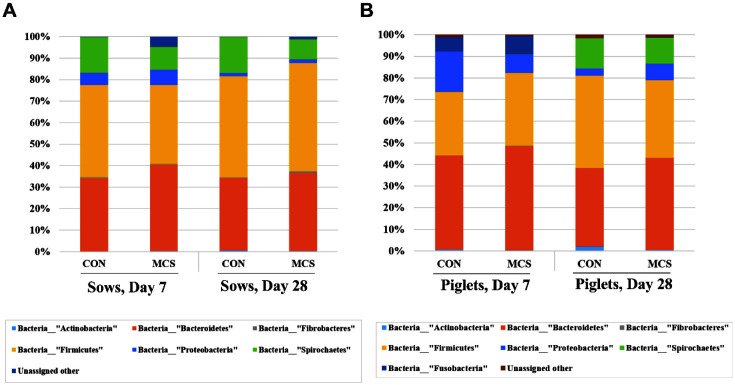
Taxonomic classification of total bacteria at phylum level retrieved from pooled DNA amplicons from feces of CON (*n* = 3) and MC (*n* = 3) groups of sows (**A**) and piglets (**B**) at day 7 and 28.

**Fig. 3:**
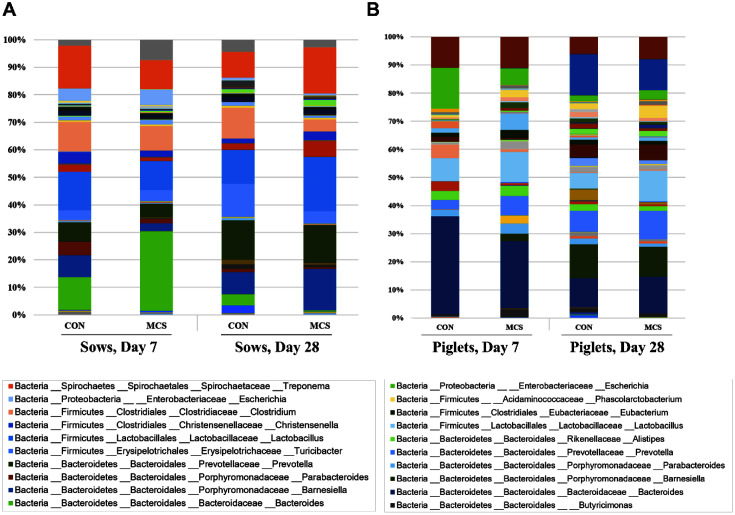
Taxonomic classification of total bacteria at genus level retrieved from pooled DNA amplicons from feces of CON (*n* = 3) and MC (*n* = 3) groups of sows (**A**) and piglets (**B**) at day 7 and 28. Top 10 of the bacterial genera that present most highly in sows or piglets are indicated in the box.

To further classify the 16S rRNA gene sequencing reads into different taxonomies, relative bacterial abundance of each sample was generated at phylum and genus levels (Figs. 2 and 3). Overall, at the phylum level, Firmicutes and Bacteroidetes were dominant in fecal samples of pigs. In sows, the proportion of Bacteroidetes was higher in the MC group than in the CON group, both during lactation (37.20% ± 0.15 in MC vs. 31.30% ± 0.078 in CON) and at weaning day (33.58% ± 0.020 in MC vs. 31.49% ± 0.037 in CON, Fig. 2A). Supplement of MC in lactating diets decreased the proportion of Spirochaetes in fecal samples from both times. At the phylum levels of piglets, the litters from MC group had more Actinobacteria than those from the CON group (0.25% ± 0.00057 in MC vs. 0.63% ± 0.00057 in CON, Fig. 2B).

Bacteroidetes produce short-chain fatty acids and create an acidic environment, which could inhibit the growth of intestinal pathogens such as pathogenic *Escherichia coli*, *Salmonella* spp., and *Clostridium* spp. [21]. Bacteroidetes are also able to digest indigestible nutrients, which in turn provides extra energy to their host [22]. With respect to the decreased abundance of Spirochaetes in the MC-treated sows, Spirochaetes has the ability to degrade fibers such as hemicellulose [23]. From these findings, it is clear that exogenous carbohydrases in the lactating sow diet induce shape changes of the intestinal microbiota.

### Dietary Carbohydrases Alter Microbial Compositions at the Genus Level in Lactating Sows and Piglets

When looking at the differences in relative abundances of bacterial genera, *Bacteroides* and *Lactobacillus* were dominant in fecal samples of sows on days 7 and day 28, respectively (Fig. 3A). With respect to the MC supplement, the proportions of *Barnesiella* (2.76% in MC vs 7.36% in CON), *Lactobacillus* (9.79% in MC vs 12.97% in CON), and *Treponema* (9.87% in MC vs 14.23% in CON) decreased with the MC treatment compared with the control at day 7 in sows, while *Christensenella* (2.94% in MC vs 1.47% in CON), *Lactobacillus* (18.34% in MCS vs 11.45% in CON), and *Treponema* (15.57% in MC vs. 8.59% in CON) increased at day 28. The treatment of MC decreased the relative abundances of *Turicibacter* (4.18% in MC vs. 11.07% in CON) and *Clostridium* (4.10%in MC vs. 10.31% in CON) in sows at day 28. Similar to sows, piglets also possessed *Bacteroides* and *Lactobacillus* as dominant fecal bacteria (Fig. 3B). The proportions of *Prevotella*, *Lactobacillus*, and *Treponema* were higher in the piglets from the MC-treated sows at both days than those from the control diet-fed sows.

**Fig. 4:**
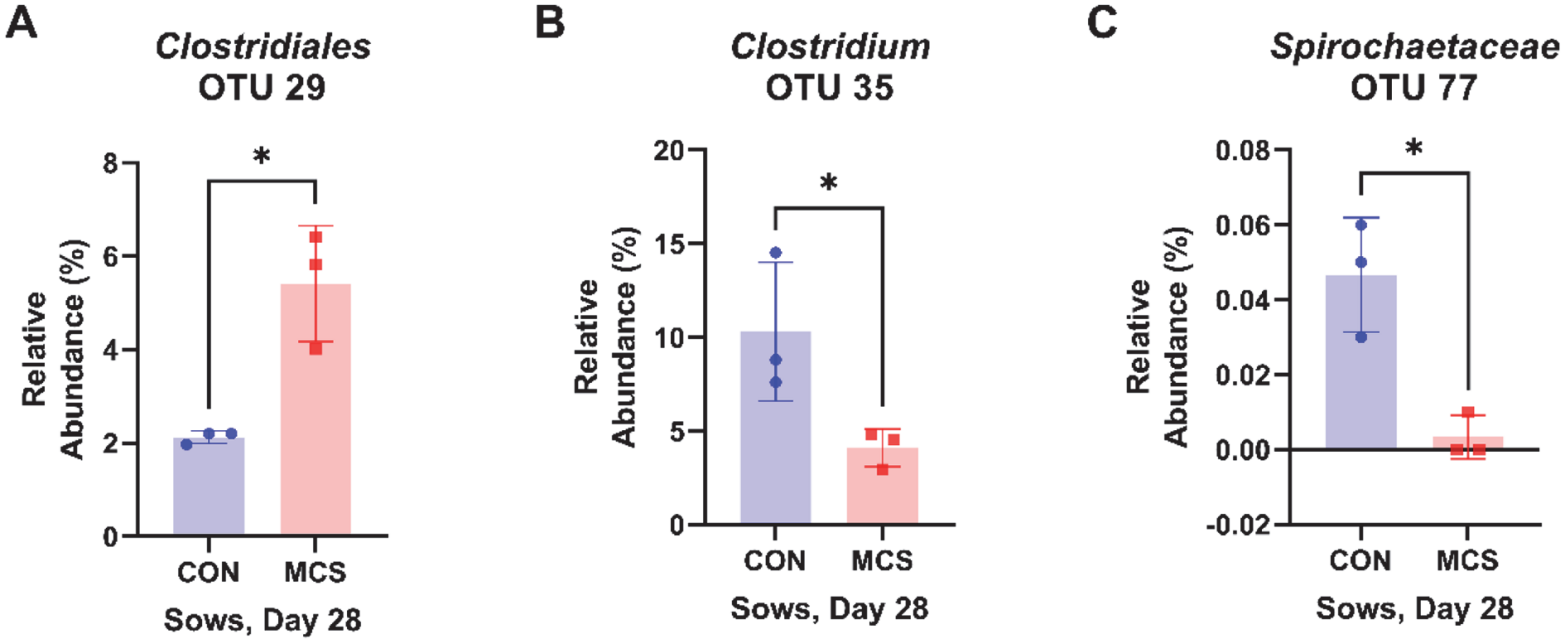
Significantly different bacterial relative abundance retrieved from pooled DNA amplicons from feces of CON (*n* = 3) and MC (*n* = 3) groups of sows at day 28. The data were analyzed by Student’s *t*-test, and statistical significance was considered at *p*-value < 0.05.

Among the various bacterial taxa present in swine fecal samples, specifically *Clostridiales* (OTUs 29), *Clostridium* (OTUs 35) and *Spirochaetaceae* (OTUs 77) showed significantly different relative abundances when treated with MC than the control sows at day 28 (Fig. 4). Especially, the MC treatment reduced the abundances of *Clostridium* and *Spirochaetaceae* at the level of 2.5-fold and 14.1-fold compared with the values of control, respectively.

*Lactobacillus* spp. or lactic acid bacterium create an acidic environment in the intestine and inhibit the growth of intestinal pathogens, conferring gut health benefits on the host [24]. Supplementation of MC is predicted to change the profile of available nutrients, mainly carbohydrate-associated fibers and oligosaccharides, in the gut [25]. It is also well known that maternal diets affect maternal milk composition and gut microbiota in offspring [26]. From the crosstalk between the gut nutrients and the milk composition, it can be speculated that MC may increase availability of milk oligosaccharides, and the increase would promote the growth of health-promoting lactic acid-producing bacteria, further resulting in better growth performance. In association with the current findings, the proportions of bacterial strains such as *Treponema* and *Prevotella* were higher with the MC treatment in the lactating sows or their piglets. *Treponema* is a genus of spiral-shaped bacteria called spirochetes and has been found in the gut microbiome of traditional rural human populations [27]. This microbe is mainly involved in the digestion of dietary polysaccharides, which are fiber, including cellulose and lignin, and the production of significant amounts of short-chain fatty acids. *Prevotella* is a gram-negative bacterium which also can use soluble fiber and is considered an important organism for biodegrading complex sugars in non-ruminant animals [28]. Taken together, the increase in available nutrient sources produced by MC would afford the ability to use the diet sources more easily while also enriching bacterial diversity. Considering the fact that they are largely involved in degrading fiber, their increase would possibly result in improvement of growth performance and immune modulation activities.

To our knowledge, the present study is the first to show the effects of dietary multigrain carbohydrase in sow diets during lactation. Our results clearly demonstrated that dietary enzymes lead to changes in species diversity and alter microbial compositions at the phylum and genus levels in lactating sows and their piglets. In order to fully understand the mechanisms of action of MC on sows and their litters, it will be necessary to further elucidate the detailed biochemical and microbial mechanisms from a productive performance and health standpoint.

## Supplemental Materials

Supplementary data for this paper are available on-line only at http://jmb.or.kr.

## Figures and Tables

**Table 1:** Number of sequences, observed diversity richness (OTUs), and diversity estimates (Chao 1, Shannon and Simpson indices) of bacteria in feces of sows and their litters at weaning point.

[Sows]	Day 7		Day 28	

	CON	MC	CON	MC

Total no. of OTUs	242.67 ± 44.30	182.33 ± 109.44	249.33 ± 17.90	231.00 ± 10.58
Chao 1	260.08 ± 46.08	194.57 ± 109.03	272.31 ± 32.62	240.44 ± 11.41
Shannon	4.64 ± 0.47	4.32 ± 1.91	5.16 ± 1.03	5.43 ± 0.17
Simpson	0.90 ± 0.04	0.83 ± 0.20	0.91 ± 0.08	0.95 ± 0.01

[Piglets]	Day 7		Day 28	

	CON	MC	CON	MC

Total no. of OTUs	93.00 ± 36.86	122.67 ± 35.10	265.33 ± 31.09	296.67 ± 31.02
Chao 1	98.88 ± 40.64	127.81 ± 34.57	285.38 ± 24.37	329.27 ± 45.01
Shannon	3.86 ± 1.10	4.68 ± 0.47	5.56 ± 0.35	5.58 ± 0.26
Simpson	0.86 ± 0.11	0.93 ± 0.02	0.95 ± 0.01	0.96 ± 0.01
