# Supplementary material for: Effects of Dietary Carbohydrases on Fecal Microbiome Composition of Lactating Sows and Their Piglets
Source: J Microbiol Biotechnol. 2022 May 4;32(6):776–82. doi: 10.4014/jmb.2203.03026 (PMC9628907; doi:10.4014/jmb.2203.03026)
Supplement: Supplementary file 1 [file jmb-32-6-776-supple.pdf]

J. Microbiol. Biotechnol.
https://doi.org/10.4014/jmb.2203.03026

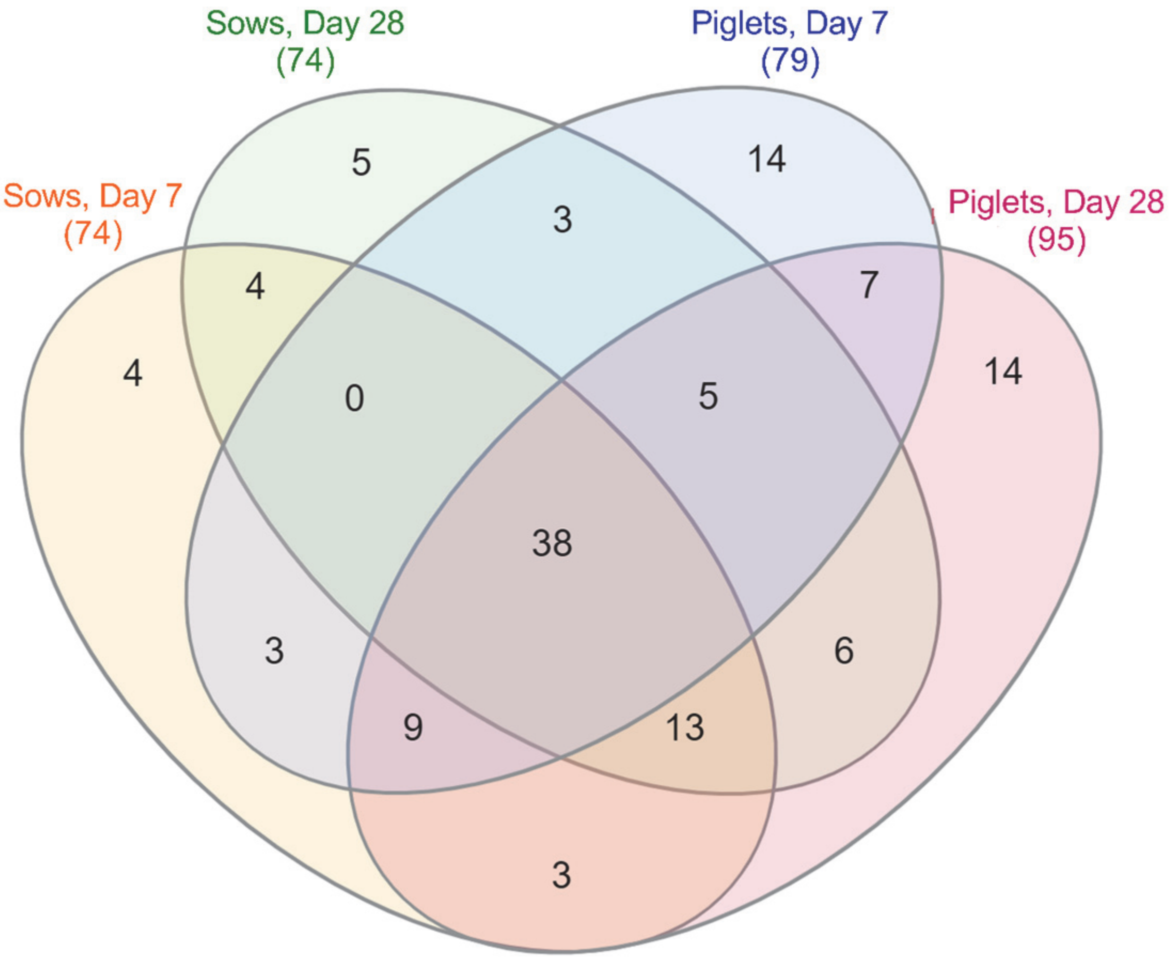


**Supplementary Fig. 1** Venn diagram illustrating the number of individual and shared genera of the sows and piglets with two feeding conditions CON and MCS at day 7 and day 28 during lactation.
